# Spatial and temporal distribution characteristics of antibiotics and heavy metals in the Yitong River basin and ecological risk assessment

**DOI:** 10.1038/s41598-023-31471-5

**Published:** 2023-03-14

**Authors:** Ke Zhao, Qian Wang, Shifeng Qian, Fengxiang Li

**Affiliations:** 1 Key Laboratory of Songliao Aquatic Environment, Ministry of Education, Jilin Jianzhu University, 5088 Xincheng Street, Changchun, 130118 People’s Republic of China; 2 Key Laboratory of Pollution Processes and Environmental Criteria at Ministry of Education, Tianjin Key Laboratory of Environmental Remediation and Pollution Control, College of Environmental Science and Engineering, Nankai University, Tianjin, 300350 China

**Keywords:** Environmental sciences, Natural hazards

## Abstract

Due to rapid socioeconomic development, antibiotic pollution and heavy metal pollution are receiving increasing amounts of attention. Both antibiotics and heavy metals in the environment are persistent and toxic, and the interactions between the pollutants create potential long-term hazards for the ecological environment and human health as mixed pollutants. In this study, the surface water of the Yitong River in Changchun was used as the research object, and the hazards associated with antibiotics and heavy metals in the surface water were assessed by analyzing the spatial and temporal distribution characteristics of antibiotics and heavy metals and by using ecological risk assessment and human health risk assessment models. The results showed that ofloxacin (OFL) and norfloxacin (NOR) varied seasonally according to the seasonal climate, with total concentrations ranging from 17.65 to 902.47 ng/L and ND to 260.49 ng/L for OFL and NOR, respectively, and from 8.30 to 120.40 μg/L, 1.52 to 113.41 μg/L and 0.03 to 0.04 μg/L for copper (Cu), zinc (Zn) and cadmium (Cd), respectively. In terms of spatial distribution, the concentration of antibiotics in the urban sections, which had intensive human activities, was higher than that in the suburban sections, while the concentration of heavy metals in the suburban sections, which had intensive agricultural operations, was greater than that in the urban section. Ecological risk evaluation showed that NOR and OFL were present in the water bodies at a high-risk level, Cd was at a low pollution level, and the heavy metal Cd was the primary pollutant associated with health risks toward for adults and children, and it was mainly at a medium risk level. Additionally, both antibiotics and heavy metals posed higher health risks for children than for adults.

## Introduction

In recent years, the coexistence of antibiotic and heavy metal pollutants has caused widespread concern. Numerous reports have shown that antibiotics and heavy metals enter water environments through domestic sewage and animal excretion and are frequently detected in different rivers and lakes^1,2^. Although trace amounts of antibiotics and heavy metals are necessary for animal growth, the presence of excessive amounts of antibiotics and heavy metals are toxic to the environment and to organisms; they accumulate and become concentrated in the environment over time, and overuse can induce the production of antibiotic-resistance genes (ARGs), metal resistance genes (MRGs) and even multidrug resistance genes in bacteria, potentially harming humans and aquatic animals through the food chain and other means^3–8^. Therefore, in our study, we evaluated the spatial and temporal distribution and ecological risk levels associated with antibiotics and heavy metals in the aqueous environment.

Most previous studies focused on individual antibiotic or heavy metal levels in water bodies^9–11^. Some researchers have shown that the surface waters in the lower reaches of the Yangtze River and in the Yitong River basin in Changchun are contaminated with both antibiotics and heavy metals to different degrees, but little research has been conducted on the extent of contamination and the toxic effects resulting from interactions between these combined pollutants in rivers and lakes^12–15^. Antibiotics and heavy metals in the environment are usually discharged into water bodies by manure, surface runoff, etc., polluting the environmental with antibiotics and heavy metals, especially during the long-term cross-use of different antibiotics and heavy metals in farms, which may induce the production of ARGs and MRGs and the growth of antibiotic resistant bacteria (ARB) and even pathogenic bacteria in water bodies^16,17^. Studies have shown that residual antibiotics interact with coexisting metal ions to form antibiotic–metal complexes (AMCs) that have altered biological activities and physicochemical properties, and these complexes are typically more toxic^18,19^. Therefore, it is important for us to investigate and study combined antibiotic and heavy metal pollution in the environment.

The Yitong River is the largest water system in Changchun, and it travels the length of the entire city. It carries the main industrial and domestic wastewaters of Changchun and is used for the important task of agricultural irrigation. With continuous improvements in the living standards of residents and with rapid development in operations such as industries, agriculture and medical care, pollution levels in the Yitong River water have increased. The Yitong River in Changchun is a typical urban river in the northern region, and the area has a harsh local climate. It experiences large temperature differences from summer to winter. Furthermore, few studies have investigated complex contamination by antibiotics and heavy metals; therefore, it is crucial to clarify the distribution and health risks associated with antibiotics and heavy metals. In this study, the spatiotemporal distribution of antibiotics and heavy metals was studied in June, August, October and December in the Yitong River, Changchun, and the potential hazards from antibiotics and heavy metals to the environment and human health were assessed by ecological risk assessment and human health assessment models. The results of the study provide a scientific basis for the integrated prevention and control of antibiotics and heavy metals and have important reference value for other rivers in the harsh climates of the northern regions.

## Methods

### Study area overview and sample collection

The Yitong River originates on the north side of the Qingdingzi Ridge of the Jilin Hadaling Mountain Range in Yitong County, flows northwest through Changchun City, and enters the Drinking Horse River east of Leaning Town in Nongan County. The watershed covers an area of 8440 square kilometers, with a river length of 342.5 km and an average river slope of 0.3‰. In the area above the Xinlizheng Reservoir in the suburbs of Changchun City is a low, mountainous and hilly area that has sparse miscellaneous woody growth and experiences soil erosion. The valley is 1 to 2 km wide, with a curved river channel and a sediment bottom with small pebbles. The area between the dam site of Lixing Reservoir and the mouth of the Xinkai River is hilly and terraced, the river valley is 5–10 km wide, the river channel is curved, and the river bottom contains fine sand and silt.

**Figure 1:**
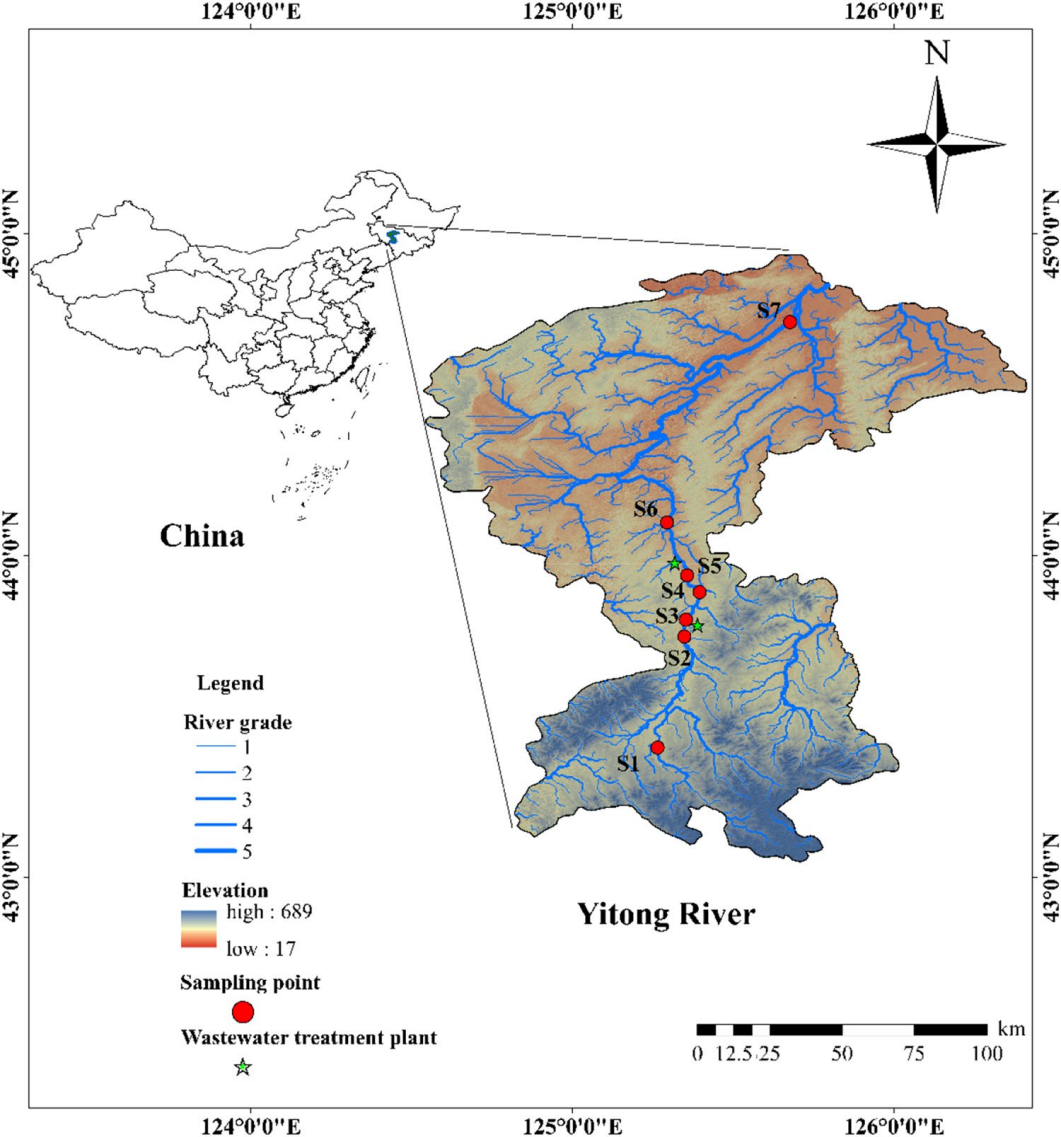
The sampling sections of Yitong River. S1 and S7 are at the upstream and downstream out of the Yitong River in Changchun City, respectively. S2, S3, S4 and S5 are the urban section points, S1, S6 and S7 are the suburban section points.

The sampling points were selected according to the distribution of towns, sewage treatment plants, villages, farmlands and river crossings along the Yitong River, and seven sampling points were set up along the river to collect fresh water samples in these seven representative river sections. Among them, S1 was in the downstream Xingguang section of the urban area of Yitong Manzu Autonomous County, which is a dense area of agricultural land. S2 was downstream of the Xinlizheng Reservoir, the main water source for Changchun City. S3 was at the confluence of the South Creek Wetland Park and the Southeast Changchun Wastewater Plant. S4 was at the Freedom Bridge, which is in a well-traveled and densely populated area within Changchun. S5 was near the Yangjiahuizi section, near the northern suburban sewage plant. S6 was at the Paulownia Bridge cross section, which is an area of dense farmland. S7 was at the cross-section of the Leaning Hill Bridge, which contains dense farmland, and the location of the sampling point cross-section is shown in Fig. 1 Surface water samples were collected at 0–50 cm depths from the Yitong River basin in June, August, October and December 2020, and the collected water samples were stored in prewashed brown glass bottles. Three-liter water samples were collected from each sampling site and stored at 4 °C with light-proof refrigeration until sample pretreatment.

### Antibiotic analysis of water bodies in the Yitong River basin

A measuring cylinder was used to accurately measure 1 L of water sample, the suspended particles in the water sample were removed by a 0.45 μm glass fiber filter membrane, 0.5 g of disodium ethylenediaminetetraacetate (Na_2_EDTA) was added, the pH was adjusted to 3 ~ 4 using 1 mol/L hydrochloric acid and ammonia, and the mixture was stirred until the salt was completely dissolved. An Oasis HLB solid phase extraction column (200 mg/6 mL) was activated with 6 mL of methanol and 6 mL of ultrapure water, and the loading rate of the extracted water samples was approximately 2 ~ 5 mL/min. The column was subsequently washed with 10 mL of ultrapure water, dried under vacuum with a negative pressure for 30 min, and eluted with 5 mL of methanol (containing 0.1% formic acid) and 5 mL of acetonitrile. The eluate was concentrated by nitrogen sparging until it was dry. Then, a 10% (v/v) methanol aqueous solution was added, the sample was redissolved to a volume of 1 mL, mixed and passed through a 0.22 μm organic filter membrane, and then it was transferred to a 2 mL sample vial and analyzed by LC‒MS/MS. The recoveries of the spiked samples were within a range of 80% ~ 115%, which met the experimental requirements. The limits of detection (LODs) for NOR and OFL were 0.01 and 0.005 ng/L, the limits of quantification (LOQs) were 0.0122 and 0.00865 ng/L, and the signal-to-noise ratios (S/N) were 3:1 and 10:1, respectively. The target antibiotics used in the experiment were the quinolone antibiotics ofloxacin (OFL) and norfloxacin (NOR), which were purchased from Shanghai Yuanye Biotechnology Co., Ltd., and all reagents were of analytical grade (purity > 95%) with a linear correlation coefficient r^2^ ≥ 0.99, which met the analytical requirements.

Analysis was performed using LC–MS/MS (3200 QTRAP LC–MS/MS system) with chromatographic separation on an Agilent InfinityLab Poroshell 120 EC-C18 (2.1 × 100 mm, 2.7-Micron, Agilent, USA) at a column temperature of 40 °C. The chromatographic separation was carried out at a flow rate of 0.1 mL/min and an injection volume of 10 μL. The flow rte was 0.30 mL/min and the injection volume was 10 μL. electrospray was used in positive ion mode (ESI+) with an electrospray source for multiple reaction detection (MRM). 0.1% (v/v) formic acid and 0.1% (v/v) methanolic solution of formic acid in water were used as mobile phases A and B, respectively. the gradient elution procedure was as follows: 90–85%A (0–2.50 min), 85–40%A (2.50–5.50 min), 40–5%A (5.50–6.00 min), 5%A (6.00–8.00 min), 5–90%A (8.00–8.10 min) and 90%A (8.10–9.00 min). Detailed parameters are shown in Supplemental Table 1.

### Analysis of heavy metals in water bodies in the Yitong River basin

The collected water samples were first filtered through a 0.45 μm cellulose acetate membrane, the suspended particles were filtered out under vacuum and high pressure, and the filtrate was collected in a 30 mL brown glass bottle. Finally, inductively coupled plasma optical emission spectrometry was used to determine the concentration of heavy metals in the water. The sample spike recovery was within a range of 95% ~ 105%, which met the experimental requirements, and the linear correlation coefficient r^2^ ≥ 0.99, which also met the requirements.

### Ecological risk and human health risk assessment of water bodies in the Yitong River basin

#### Ecological risk assessment of antibiotics

The ecological risk assessment of antibiotics usually involves the use of risk quotient (RQ) to determine whether a given concentration of a chemical contaminant is potentially harmful and characterizes the potential ecological risk from multiple contaminants in aquatic ecosystems^20^. According to the RQ value, risk can be categorized into the following 3 levels, namely, RQ ≤ 0.1 (low risk), 0.1 ≤ RQ ≤ 1 (medium risk) and RQ ≥ 1 (high risk), and the ecological risk parameters are shown in Supplemental Table 2. The calculation formula is as follows^21^:

1 $$PENC={EC}_{50}({LC}_{50})/AF$$

2 $$RQ=MEC/PNEC$$

where MEC and PNEC are the measured ambient concentration (ng/L) and the no-effect predicted concentration, respectively. The semilethal concentration L(E)C_50_ and evaluation factor AF were calculated. Most current studies on the ecological risk evaluation of antibiotics have focused on environmental risks arising from individual antibiotics, but some studies have shown that the toxic effects of antibiotics are enhanced when multiple antibiotics are simultaneously present in waters^22,23^.

#### The Nemerow integrated pollution index method for heavy metals

Multiple types of heavy metals often coexist in water bodies, and the Nemerow integrated pollution index method is a common method for evaluating heavy metal pollution in water bodies. It can reflect the current status of heavy metal pollution in water bodies and the different contributions of various heavy metals to the composite pollution effect, and it can screen the main pollutants^24^. The single-factor pollution index is divided into four levels, namely, P_i_ ≤ 1 for no pollution, 1 < P_i_ ≤ 2 for low pollution, 2 < P_i_ ≤ 3 for medium pollution, and P_i_ > 3 for high pollution. The multifactor pollution index is divided into four levels, namely, P_n_ ≤ 0.7 for no pollution, 0.7 < P_n_ ≤ 1 for low pollution, 1 < P_n_ ≤ 2 for medium pollution, and P_n_ > 2 for high pollution.

Single-factor pollution index:

3 $${P}_{i}={C}_{i}/{S}_{i}$$

Multifactor composite pollution index:

4 $${P}_{n}=\sqrt{\frac{{max({P}_{i})}^{2}+ave{({P}_{i})}^{2}}{2}}$$

where *Ci* is the measured concentration of heavy metal *i*, *Si* is the corresponding environmental standard value of heavy metal *i* with reference to the V standard limit in Supplemental Table 3 as the critical value, *max(P*_*i*_*)* is the maximum value of the single-factor pollution index of heavy metals, and *ave(P*_*i*_*)* is the average value of the single-factor pollution index of each metal.

#### Antibiotic human health risk assessment

Since NOR and OFL were detected in the Changchun Yitong River Basin at seven points to various extents, human health risk assessment was conducted for both antibiotics, and the risk entropy (RQ_H_) of antibiotics to human health was calculated based on the acceptable daily intake (ADI) of antibiotics in humans. The calculation formula is as follows^25^:

5 $${RQ}_{H} = MEC/DWEL$$

where RQ_H_ is the entropy value of the health risk for a single antibiotic, MEC is the measured concentration, and DWEL is the drinking water equivalent value.

The formula for calculating the drinking water equivalent value is as follows:

6 $$DWEL=\mathrm{ADI}\times \mathrm{BW}\times \frac{\mathrm{HQ}}{\mathrm{DWI}\times \mathrm{AB}\times \mathrm{FOE}}$$

where ADI is the average acceptable daily intake, and the values for NOR and OFL are 11.4 and 3.2 μg/(kg day), respectively^25^. BW is the body weight per capita. HQ is the highest risk, calculated to be 1. DWI is the daily water intake. AB is the gastrointestinal absorption rate, calculated to be 1. FOE is the exposure frequency 350 d/a, calculated to be 0.96. The values of BW and DWI for different age groups are shown in Supplemental Table 4. Risk level: RQ_H_ > 1 indicates that the risk from antibiotics to human health is high. When 0.1 < RQ_H_ < 1, it indicates medium risk. When RQ_H_ < 0.1, it indicates low risk^26^.

#### Heavy metal human health risk assessment

According to the evaluation method and health evaluation model issued by the U.S. Environmental Protection Agency, humans are mainly exposed to heavy metals in water bodies through drinking water and dermal contact. The acceptable risk level for hazardous substances is specified in the range of 1E−06 to 1E−04; less than 1E−06 indicates that the risk is not significant, between 1E−06 and 1E−04 indicates that there is a risk, and higher than 1E−04 indicates that there is a more significant risk. The potential total health risk (R) from metals can be divided into two categories: carcinogenic risk (R^c^) and noncarcinogenic risk (R^n^)^27^.

Carcinogenic risk through the drinking water route:

7 $${R}_{i}^{c}=\frac{{ADD}_{i}\times S{F}_{i}}{L}$$

where ${R}_{i}^{c}$ is the average annual health risk (a − 1) for an individual from the carcinogenic metal i through the drinking water route. *SFi* is the carcinogenic slope factor of the carcinogenic metal i, and the value for Cd is 6.1 mg/kg/day.

When ${R}_{i}^{c}$ was calculated to be greater than 0.01, it was considered high exposure:

8 $${R}_{i}^{c}=\frac{1-{\mathrm{exp}(-ADD}_{i}\times S{F}_{i})}{L}$$

Noncarcinogenic risk modeling for the drinking water route:

9 $${R}_{i}^{n}=\frac{{ADD}_{i}}{{RfD}_{i}\times L}\times {10}^{-6}$$

10 $${ADD}_{i}=\frac{{C}_{i}\times IR\times EF\times ED}{BW\times AT}$$

where ${R}_{i}^{n}$ is the average annual health risk to an individual from the noncarcinogenic metal element i through the drinking water route, a^−1^; and *Rfd*_*i*_ is the reference dose of a pollutant *i* under a certain exposure route, which has values of 0.005 and 0.3 mg/kg/day for copper (Cu) and zinc (Zn), respectively. *ADDi* is the average daily exposure to a pollutant, mg/kg/day. *Ci* is the concentration of a pollutant in water, mg/kg/day; *IR* is the intake of drinking water, with an average of 2.2 L/day for adults and 1 L/day for children. *BW* is the body weight, with an average of 60 kg for adults and 25 kg for children. *EF* is the exposure frequency of 365 day/a. *ED* is the exposure duration, the duration of carcinogenic exposure is 70 a, and the duration of noncarcinogenic exposure is 35 a. *AT* is the average exposure frequency of 365 day/a. *ED* is the exposure duration, the duration of carcinogenic exposure is 70 a, and the duration of noncarcinogenic exposure is 35 a. The average exposure time was 25,550 day for carcinogenic exposure and 12,775 day for noncarcinogenic exposure. *L* is the average human life expectancy, and the actual value for the study area was calculated based on the average life expectancy of 77.93 year in Changchun City in 2022. The recommended average life expectancy was calculated based on 78 a^28,29^.

#### Mixed antibiotic-heavy metal toxicity assessment

Antibiotics and heavy metals exist in mixed forms in the environment for long periods of time, and the evaluation of mixed toxicity is an issue of great interest in current research. The determination method usually involves a combination of the toxicity unit method and the summation index method, and determination of the combined effect of the mixture is based on M = 1, AI = M − 1, M < 1, AI = 1/(M − 1), M > 1, AI = 1 − M. AI is the summation index, and the model determines the joint action of the mixture as follows: when AI = 0, the joint action of the mixture is the sum; When AI < 0, the combined action of the mixture is antagonistic. When AI > 0, the combined action of the mixture is synergistic and is calculated with the following equation^30^:

Toxicity unit method:

11 $${\mathrm{TU}}_{\mathrm{i}}=\frac{{\mathrm{C}}_{\mathrm{i}}}{{\mathrm{EC}}_{50\mathrm{i}}}$$

Summation index method

12 $$\mathrm{M}=\sum {\mathrm{TU}}_{\mathrm{i}}$$

where TU_i_ is the i-th substance toxicity unit. C_i_ is the i-th substance concentration (mg/L). EC_50i_ represents the i-th substance semilethal concentration in a single system, see Supplemental Table 2. The toxicity unit method treats the i-th substance semilethal concentration in a single system as 1 toxicity unit, and M is the mixed toxicity unit.

### Data analysis methods

Water quality indicators, heavy metals and antibiotic concentrations were statistically and graphically presented using Excel and Origin 2021b software. Correlation analysis was performed using IBM SPSS Statistics 26 software. Map created using ArcGIS 10.6 (https://www.esri.com/en-us/arcgis/products/arcgis-desktop/overview).

## Discussion

### Spatial and temporal distribution characteristics of antibiotics and heavy metals in the Yitong River

#### Spatial and temporal distribution characteristics of antibiotics

**Figure 2:**
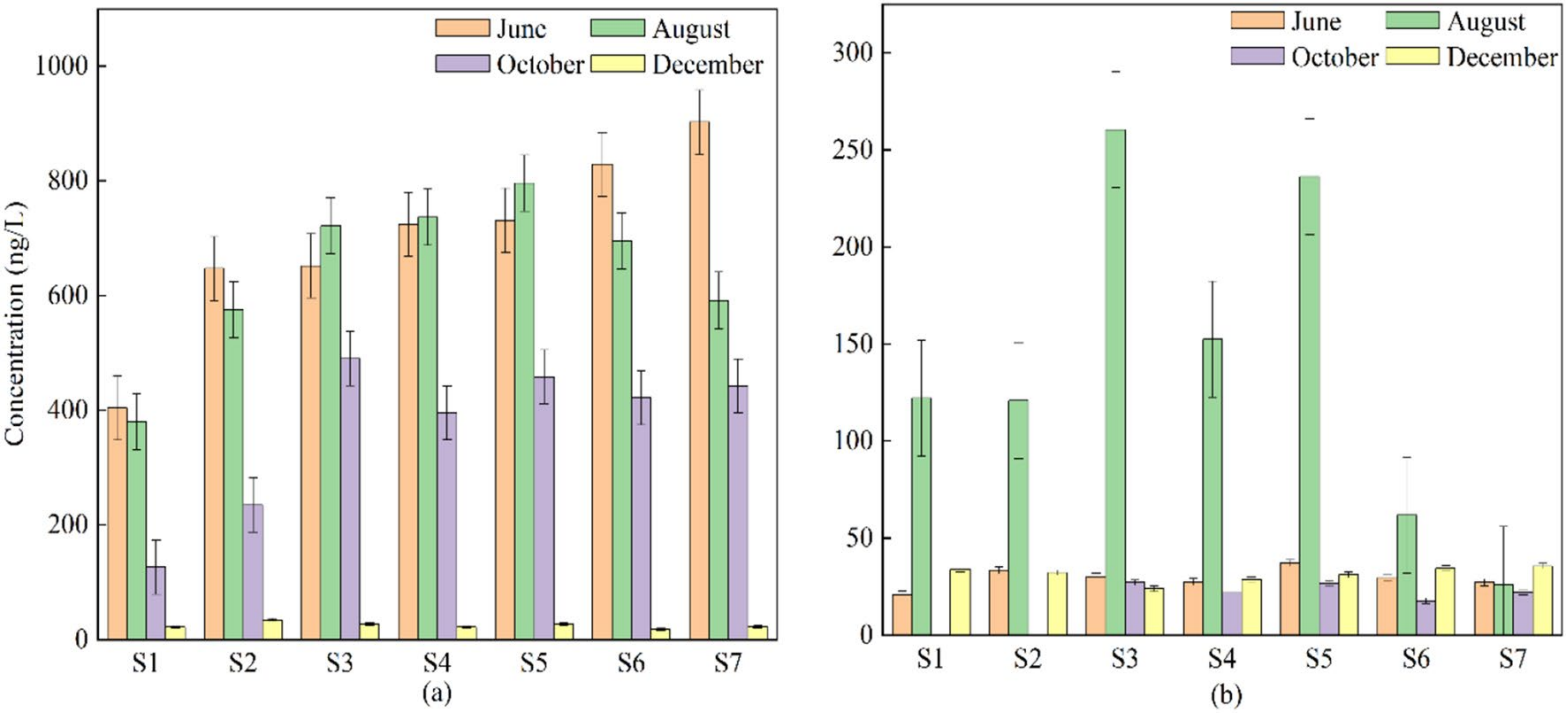
Spatial and temporal distribution characteristics of antibiotics in surface water. (**a**, **b**) are histograms of NOR and OFL concentrations in the Yitong River surface water in June, August, October and December.

The spatial distribution and seasonal variation of NOR and OFL antibiotics in surface waters at the seven sampling sites in the Yitong River, Changchun, are shown in Fig. 2. In terms of seasonal distribution characteristics, the concentrations of the quinolone antibiotics NOR and OFL were higher in summer than in winter, with decreases of 96.49% and 77.55% in NOR and OFL concentrations, respectively, from June to December 2021. According to the Jilin Provincial Meteorological Bureau, the province received 688.4 mm of precipitation in 2021, with a significant increase in precipitation from summer to winter. Additionally, flooding occurred due to discharges from the new Licheng Reservoir upstream of the Yitong River in Changchun city starting in October, leading to an increase in river flow. Changchun City is in a northern cold region, temperatures plunge from summer to winter, and the river surface in the winter has a 20 ~ 440 cm thick ice layer, which may also lead to a reduction in the surface water antibiotic concentration; thus, the summer antibiotic concentration might be higher than the winter antibiotic concentration.

Based on spatial analysis, both the antibiotics NOR and OFL were detected to varying degrees in the urban sections (S2, S3, S4, S5) and suburban sections (S1, S6, S7), and NOR had a 100% detection rate in both the urban and suburban sections. The detection rate for OFL reached 93.75% in both the urban and suburban sections. The measured NOR concentrations in the urban sections were higher than those in the suburban sections, with concentration levels ranging from 21.8 to 796.04 ng/L and 17.65 to 902.47 ng/L, respectively. OFL concentrations in the urban sections were lower than those in the suburban sections, with concentration levels of ND ~ 260.49 ng/L and ND ~ 122.08 ng/L, respectively.

According to a survey, quinolone antibiotics have been detected in various rivers and lakes in China and other countries, as shown in Table 1. The concentration of NOR in the surface water of the Yangtze River Delta, Han River Plain, Dongjiang River and Beijiang River in China ranged from ND to 134.2 ng/L. The concentration of NOR in the surface waters of the Northern Basin and Seine River Estuary in Pakistan ranged from 0 to 8.39 ng/L. In comparison, the NOR concentrations reported in the surface waters of the Yitong River in Changchun by Chinese and foreign researchers were at a high level. The concentrations were similar to those in the Mediterranean River (maximum concentration of 936 ng/L) and the surface waters of southeast Queensland (maximum concentration of 1150 ng/L). The concentrations of OFL in the surface waters of the Yangtze River Delta, the Han River Plain and the Beijiang River in China ranged from ND to 135.1 ng/L^19,31,32^. The concentrations of OFL in the surface waters of the northern basin and the inner estuary of the Seine River in Pakistan ranged from 0 to 6 ng/L, and these were roughly similar to those in the surface waters of the Yitong River in Changchun but much lower than those in the Red River in India^33–35^.

**Table 1:** Comparison of antibiotic and heavy metal concentrations in surface water of rivers and lakes at home and abroad.

Rivers and lakes	Antibiotic concentration (ng/L)			Rivers and lakes	Heavy metal concentration (μg/L)			
	NOR	OFL	literature		Cu	Zn	Cd	literature
Yitong River, Changchun City	902.47	260.49	This study	Yitong River, Changchun City	44.92	25.89	1.88	This study
Surface water in the Yangtze River Delta	48.39	18.95	^38^	Yangtze River	10.7	9.4	4.7	^39^
Beijiang River	35.3	36.7	^31^	Yellow River	36.27	52.46	23.19	^40^
Surface water of the Han River Plain	134.2	135.1	^32^	Han River	7.76	–	3.21	^41^
Yangtze River and Jialing River	–	26.81	^10^	Yangtze Estuary	2.89	9.53	0.092	^42^
Fenhe and Yellow rivers	14.89	7.68	^43^	Ajay River Basin	80	29	30	^44^
Red River Delta	–	4130.0	^33^	Pearl River	8.24	–	0.06	^45^
Northern Basin of Pakistan	38.0	96.0	^34^	Catalan river basins	1.3	1.9	2.9	^46^
Mediterranean river	936.0	–	^47^	Tigris River	13.25	52.75	2.65	^48^
Seine River	48.39	18.95	^35^	Lich River	4.5	51.1	2.65	^49^
Watersheds of South-East Queensland	1150.0	–	^50^	Sava River	0.54	2.27	0.011	^51^

"–" indicates that the class of substances were not detected.

Overall, NOR and OFL concentrations in the surface waters in the Yitong River basin in Changchun are at higher levels, and water pollution is more severe. The residual NOR and OFL antibiotic concentrations may be related to the population density and the distribution of industries such as biopharmaceuticals. The production of pharmaceuticals in industries and the use of drugs in hospitals result in the production of large volumes of wastewater that contain large amounts of antibiotics, and these wastewaters cannot be completely treated in sewage plants. The secondary effluent of treatment plants discharge antibiotic residues directly into nearby water bodies, and antibiotic concentrations ultimately increase in the waters^36^. Therefore, it is necessary to further strengthen awareness of environmental protection while optimizing and improving water treatment processes and technologies to enhance the level of water pollution management.

#### Spatial and temporal distribution characteristics of heavy metals

**Figure 3:**
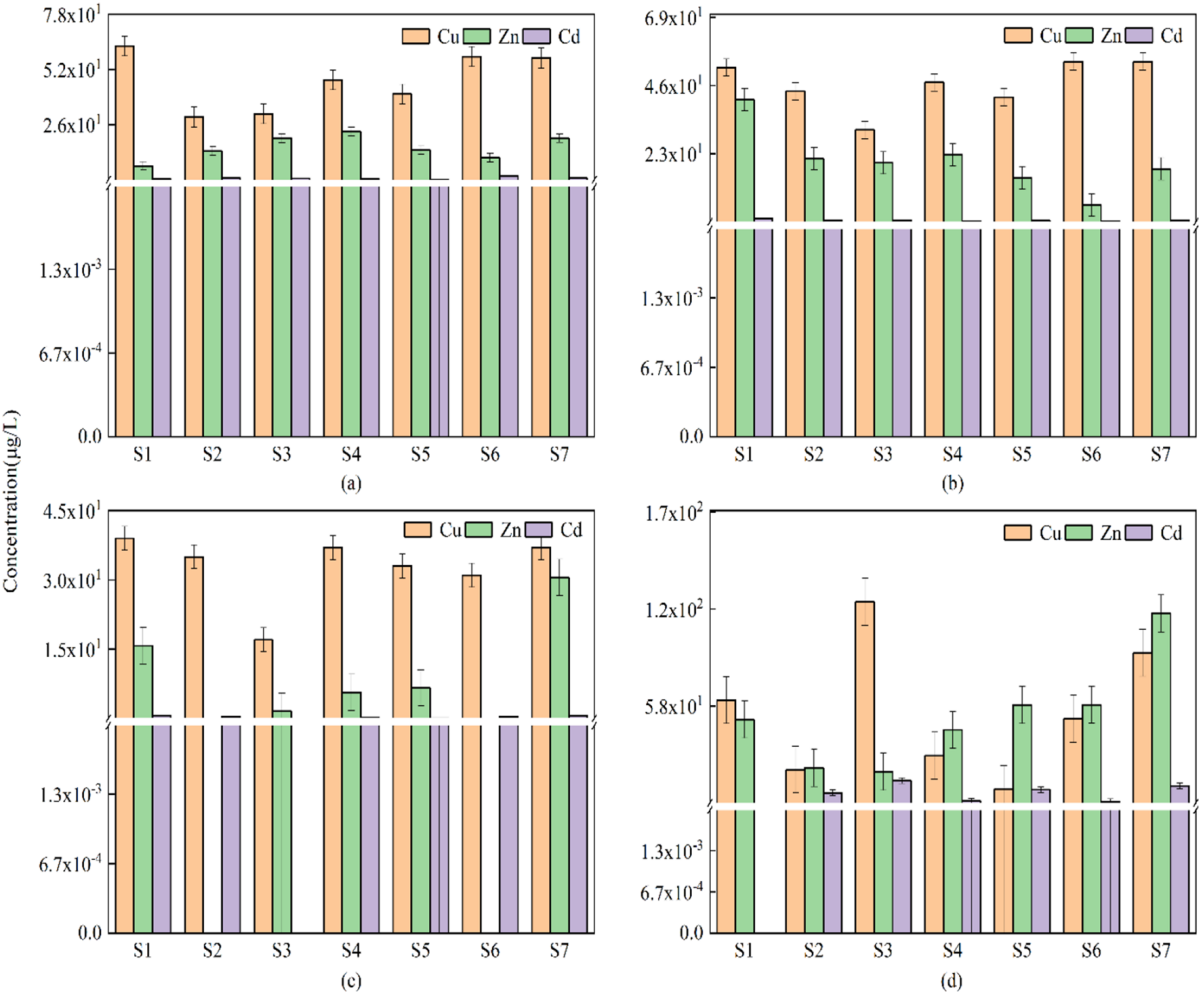
Spatial and temporal distribution characteristics of heavy metals in surface water. (**a**–**d**) are the histograms of heavy metal concentrations in the surface water of Yitong River basin in June, August, October and December, respectively.

The results of the analysis of three heavy metals, Cu, Zn and Cd, in the Yitong River basin in Changchun are shown in Fig. 3. The detection rate of Cu was 100%, and the detection rate for both Zn and Cd was 92.86%. The average concentrations (μg/L) of the three heavy metals were in the following order: Cu (44.92) > Zn (25.89) > Cd (1.88). Compared with the domestic Environmental Quality Standards for Surface Water (GB3838-2002), only the Zn level was within the Class I water standard, and the Cu and Cd levels fell within the Class II water standard. The surface water quality standards for Cu, Zn and Cd in Romania, which is at the same latitude, exceed the Class V water quality standards in China. The concentrations of heavy metals in the surface waters of other rivers in China and countries are shown in Table 1. The heavy metals in the water bodies of the Yitong River in Changchun City were in the middle level, which has a range of values that is generally higher than that of the rivers in China and other countries, but the concentration of Zn was lower than that of the Yellow River (Gansu section) in China, the Tigris River in Iran and the Lich River in Vietnam. The concentrations of Cd in the water column were approximately similar to those of the Catalan River basins in Spain, the Tigris River in Iran, and the Lich River in Vietnam. The rainfall in Changchun increased every month starting in June 2021, which led to a gradual decrease in Cu, Zn and Cd until December when the concentration of heavy metals in the water bodies of the Yitong River in Changchun increased.

The spatial distribution characteristics show that the concentration patterns of Cu, Zn and Cd were similar, and their concentrated areas were mainly at points S1, S6 and S7, followed by points S3, S4 and S5, which may be because points S1, S6 and S7 were in an agricultural production area, and some studies have shown that antibiotics and heavy metals are commonly used as feed additives in the livestock and poultry farming industry, which creates a typical environment for mixed antibiotic and heavy metal pollution in and around farms^37^. The points S3, S4 and S5 points were largely influenced by urban domestic sewage and industrial production. Therefore, the spatial and temporal distribution patterns of heavy metals in the Yitong River in Changchun were closely related to climatic causes, production levels and human social activities in Changchun.

### Correlation of antibiotics, heavy metals and water quality conditions

**Figure 4:**
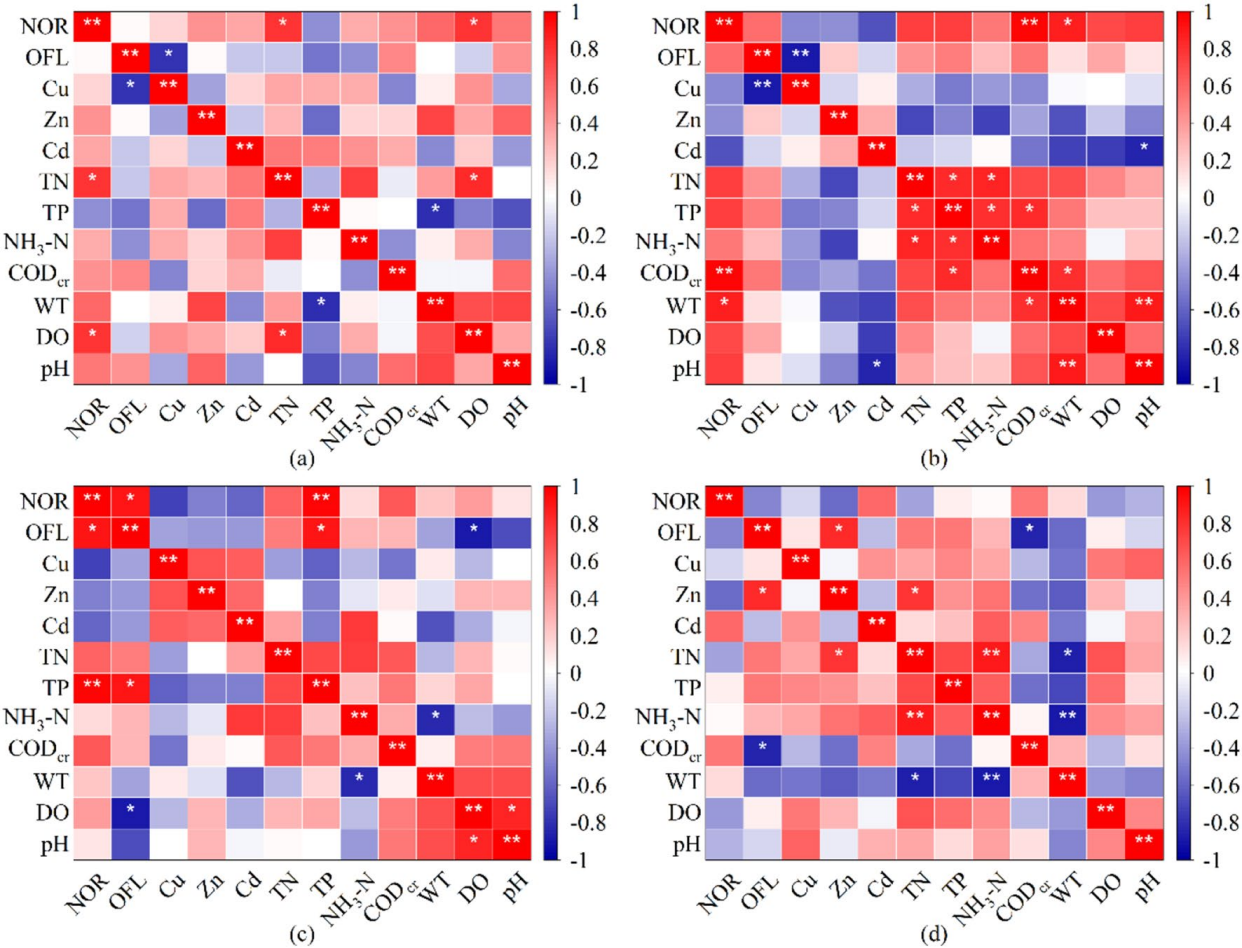
Correlation analysis of antibiotics, heavy metals and water quality conditions. (**a**–**d**) are heatmaps of correlation analysis for June, August, October and December, respectively. P < 0.05 for *, P < 0.01 for **.

To investigate the influence of water quality indicators and heavy metals in water samples on the spatial and temporal distribution characteristics of antibiotics, the water quality conditions were examined, and these are shown in Supplemental Fig. 1. Correlation analysis of antibiotics, heavy metal ions and water quality conditions was performed by Spearman’s analysis, as shown in Fig. 4 NOR with TN were significantly and positively correlated (P < 0.01) in the Yitong River water column in August, but there was no significant correlation during the rest of the month, probably because of the large difference in temperature from summer to winter in Changchun City; the area experiences severely cold temperatures in winter, and the measured water temperature in the field was in the range of 0 ~ 27 °C, and these low temperatures are more suited for bacterial survival. In winter, humans, livestock and poultry are prone to contracting viral colds and other infectious diseases, and the use of drugs for humans and animals increase the detection rate and concentration levels of antibiotics in the aquatic environment, which endangers the health of humans and animals in the long run. In addition, NOR showed a significant positive correlation with TP, TN, CODcr and DO (P < 0.05, P < 0.01), and OFL showed a significant positive correlation with TP (P < 0.05) and a negative correlation with DO (P < 0.01) in the water bodies of the Yitong River. This suggests that the NOR, OFL and nutrients present in the water may have originated from domestic wastewater discharge. Aerobic microorganisms proliferate and degrade antibiotics at high DO concentrations, which results in lower antibiotic concentrations; in contrast, low DO environments contain high antibiotic concentrations. NOR and OFL showed significant correlations with water quality indicators in December 2021, with OFL showing a negative correlation with CODcr, suggesting that water quality indicators are not the only factors determining antibiotic contamination in the Yitong River in Changchun, but that the physicochemical properties of the compounds, environmental conditions, and seasonal climate are also key factors influencing antibiotic availability and ecological risk in the river^52–54^.

In the surface water of the Itong River, the levels of antibiotics and heavy metals were analyzed by the t-test for significant differences, which showed that there was a significant difference between NOR and heavy metals, while no previous difference was observed between OFL and heavy metals, as shown in Supplemental Fig. 2. In August and October the antibiotic OFL showed a negative correlation with Cu (P < 0.05). In the surface waters of the Yitong River in August, NOR showed a significant negative correlation with Zn and Cd (P < 0.05, P < 0.01), and OFL showed a significant negative correlation with Cu (P < 0.01), but there was no significant correlation during the rest of the month. These correlations may have arisen because the antibiotics present in the water bodies were influenced by heavy metals, and strong interactions occurred between antibiotics and heavy metals, resulting in low antibiotic concentrations in environments with high concentrations of heavy metals and low levels of interactions between the two and high antibiotic concentrations in environments with low concentrations of heavy metals. Moreover, antibiotics and heavy metals remain in water bodies for long periods of time, and QNs interact with heavy metals such as Fe^2+^, Co^2+^, Cu^2+^ and Ni^2+^ to form AMCs, which are more persistent and toxic than the parent compounds. These antibiotic metal complexes are usually more potent than the individual antibiotics and heavy metals and have stronger growth inhibitory activity against Scenedesmus obliquus^18^. Although there is no apparent risk to humans from combined antibiotic and heavy metal contamination today, the risk from antibiotic metal complexes resulting from complexation of antibiotics with heavy metals under different conditions to humans and animals remains uncertain and requires careful consideration.

### Antibiotic and heavy metal ecological risk assessment and human health risk assessment

#### Ecological risk assessment of antibiotics

**Figure 5:**
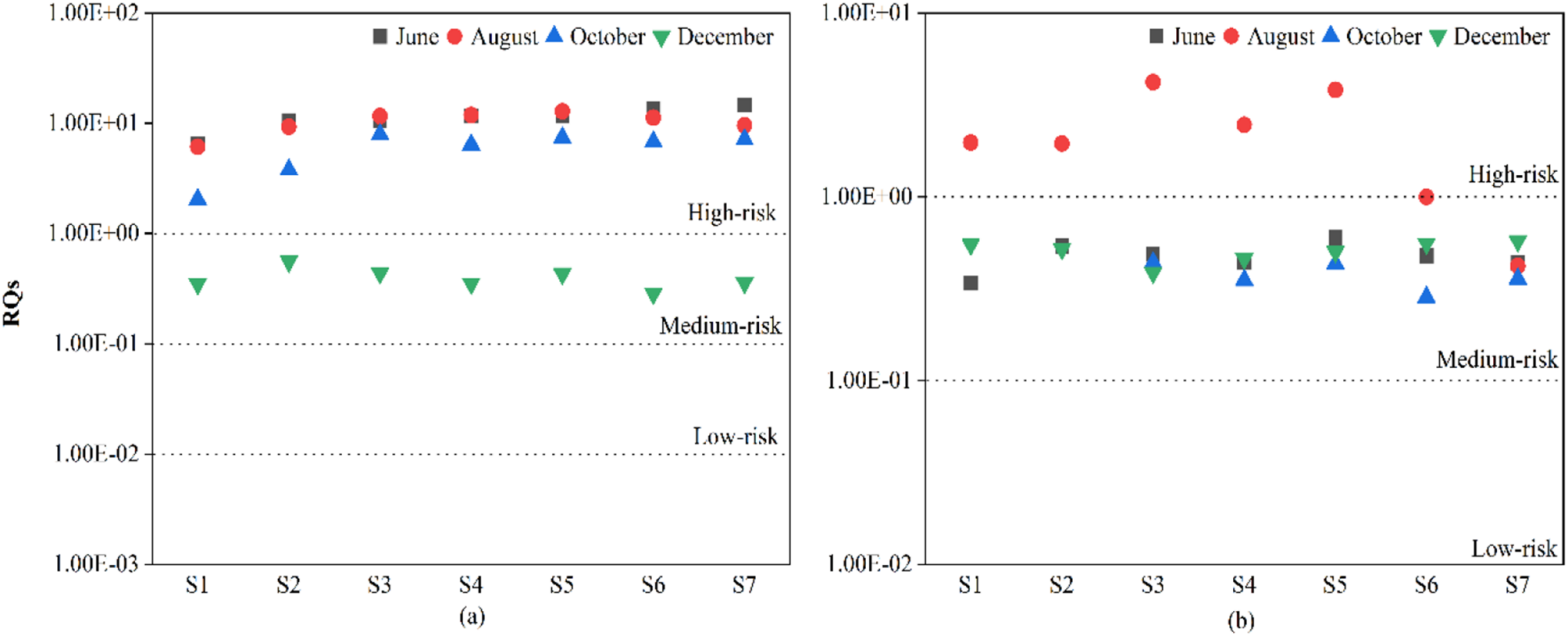
Ecological risks of NOR and OFL in Yitong River, Changchun. (**a**, **b**) represent the ecological risks of antibiotic NOR and OFL, respectively. Low risk RQ < 0.1, Medium risk (0.1 < RQ < 1), High risk (RQ > 1).

Based on the ecological risk assessment of antibiotics, the ecological risk entropy values of antibiotics at seven sampling sites in the Yitong River, Changchun, were calculated. As seen in Fig. 5, NOR and OFL present medium and high risk levels for Microcystis aeruginosa. In terms of seasonal distribution characteristics, the analysis of the ecological risk RQ values for surface water of the Yitong River in June, August, October, and December 2021 indicated that the ecological risk (RQ > 1) of NOR in the first three months was high. The order of the RQ values for different months from lowest to highest was December (0.3958) < October (5.9182) < August (10.3592) < June (11.2666). The ecological risk (0.1 < RQ < 1) from OFL in June, October and December 2021 was medium risk, and the ecological risk in August was high risk, except at points S6 and S7, which showed medium risk.

In terms of the spatial distribution characteristics, the order of RQ values for the ecological risk from NOR at each sampling point was in the following order from low to high: S1 (3.7538) < S2 (6.0152) < S4 (7.5724) < S6 (7.9149) < S3 (7.6249) < S7 (7.8981) < S5 (8.1152), while the order of RQ values for the ecological risk from OFL at each sampling point was in the following order from low to high: S7 (0.4473) < S6 (0.5788) < S4 (0.9281) < S4 (0.9523) < S2 (1.002). in descending order were S7 (0.4473) < S6 (0.5788) < S4 (0.9281) < S1 (0.9523) < S2 (1.0027) < S5 (1.3361) < S3 (1.3779). Both NOR and OFL had high ecological risk RQ values at points S3 and S5, which may have been due to the proximity of points S3 and S4 to the Southeast Changchun Wastewater Plant and the outlet of the Beijiao Wastewater Plant. Research shows that because antibiotics are widely used in many fields such as medicine production, animal husbandry, aquaculture and human medicine and traditional wastewater treatment technologies are not effective in removing them, the secondary effluent of different wastewater treatment plants have different degrees of antibiotic residues present, and these are discharge into the ecological environment during different seasons^36^. Overall, even trace levels of antibiotics (ng levels) pose some degree of ecological risk to sensitive organisms in the water column, particularly algae in the water column. At the same time, there are many kinds of antibiotics in the aquatic environment, and the toxicities of the different antibiotics are different, so it is important to manage and control the use of antibiotics to safeguard the ecological environment and human health.

#### Heavy metal ecological risk assessment

The risk of environmental pollution from the three heavy metals in the water bodies of the Yitong River was assessed using the Nemerow integrated pollution index method, as shown in Table 2. The total range of the single factor evaluation index Pi for the three heavy metals was between 0.0015 and 2.6400, with the Pi range of Zn between 0.0015 and 0.1134; these belonged to the nonpollution level. The range of the single factor evaluation indices Pi for Cu and Cd was between 0.0830 and 1.2040 and 0.0052 and 2.6400, respectively, where Cu and Cd were at low pollution levels at points S3 and S7 in December. The results suggest that the potential contributors to the heavy metal pollution of the Yitong River in Changchun were Cu and Cd, which may be due to the high population density and high traffic levels at point S3, which is influenced by human activities. At the same time, point S7 is near livestock and agricultural operations and has more truck traffic, and the Cd present in the environment enters the surface waters. The range of the Mero composite index Pn varied in different months, with Pn ranging from 0.2343 to 0.4742 in June, 0.2430 to 0.4298 in August, 0.1346 to 0.3032 in October, and 0.2343 to 2.0769 in December. Among them, point S2 (0.9121) was at a low pollution level, point S7 (1.5913) was at a medium pollution level, point S3 (2.0769) was at a high pollution level, and in June, August and October these points showed a nonpolluted level. Thus, the results indicate that the heavy metal pollution in the Yitong River Basin in Changchun city showed a decreasing trend in heavy metal pollution levels with seasonal climate changes, such as increased rainfall and larger surface runoff and had a 28.5% decrease in the Nemerow Composite Pollution Index from June to October. Additionally, since Changchun City experiences severely cold winters, the temperature is extremely low in December, the lake freezes, and the river flow decreases, which leads to heavy metal enrichment, thus causing potential harm to the aquatic environment.

**Table 2:** Heavy metal pollution index.

Pollution index	Month	Heavy metals	Sampling points						
			S1	S2	S3	S4	S5	S6	S7
P_i_	June	Cu	0.6300	0.2954	0.3100	0.4700	0.4033	0.5800	0.5731
		Zn	0.0065	0.0134	0.0196	0.0226	0.0139	0.0104	0.0196
		Cd	0.0532	0.1416	0.1150	0.0518	0.0052	0.3256	0.1420
	August	Cu	0.5200	0.4400	0.3100	0.4700	0.4200	0.5400	0.5400
		Zn	0.0412	0.0214	0.0199	0.0226	0.0149	0.0057	0.0179
		Cd	0.2314	0.1094	0.1150	0.0518	0.0766	0.0524	–
	October	Cu	0.3900	0.3500	0.1700	0.3700	0.3300	0.3100	0.3700
		Zn	0.0157	–	0.0015	0.0056	0.0066	–	0.0306
		Cd	0.1290	0.0828	–	0.0212	0.0076	0.0670	0.1128
	December	Cu	0.6150	0.1990	1.2040	0.2820	0.0830	0.5030	0.8990
		Zn	0.0498	0.0208	0.0186	0.0437	0.0586	0.0587	0.1134
		Cd	–	1.2000	2.6400	0.1962	1.5600	0.1288	2.0120
P_n_	June		0.4742	0.2343	0.2430	0.3563	0.3021	0.4635	0.4407
	August		0.4124	0.3390	0.2430	0.3563	0.3205	0.4070	0.4298
	October		0.3032	0.2910	0.1346	0.2778	0.2470	0.2565	0.2883
	December		0.4943	0.9121	2.0769	0.2343	1.1737	0.3911	1.5913

"–" indicates that the class of substances were not detected.

P_i_ is the single factor evaluation index for Cu, Zn and Cd in June, August, October and December.

P_n_ is the Nemero composite index for heavy metals in June, August, October and December.

### Human health risk assessment

**Figure 6:**
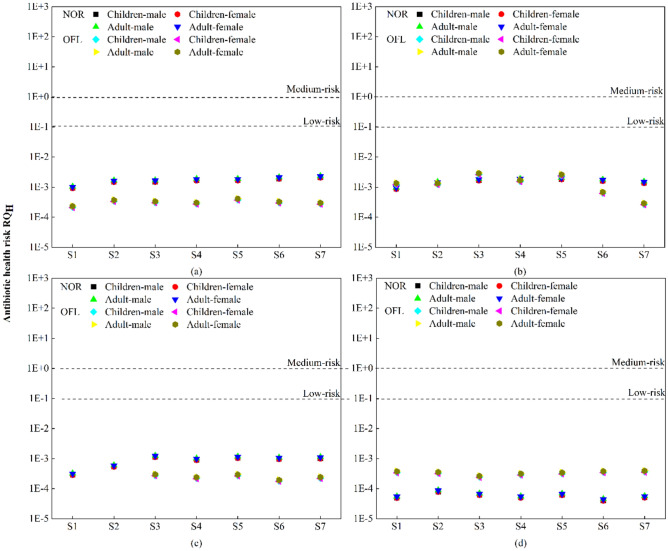
Risk assessment of antibiotic human health. (**a**–**d**) are human health risk assessments for NOR and OFL in June, August, October and December. RQ_H_ > 1 is high risk, 0.1 < RQ_H_ < 1 is medium risk, RQ_H_ < 0.1 is low risk.

Since the Yitong River runs through Changchun city, which has many densely populated areas, the risk assessment of antibiotics and heavy metals in the environment is based on the risk assessment methods of antibiotics for human health (RQ_H_) and heavy metals for human health (noncarcinogenic R^c^ and carcinogenic R^n^). The risk from antibiotics to human health (RQ_H_) was calculated to obtain the entropy value for the health risk at the 7 sampling sites during different months, as shown in Fig. 6. The results show that the human health risk RQ_H_ values for NOR and OFL in the Yitong River in Changchun were less than 0.1, which indicates a low risk level. In terms of seasonal changes, NOR and OFL showed a trend that had lower human health risks during summer than in winter. Furthermore, the mean values of NOR for the entropy of human health risk RQ_H_ for adults and children were 1.983E−03 and 2.203E−03, respectively, and the mean values of OFL for the entropy of human health risk RQ_H_ for adults and children were 1.122E−03 and 1.246E−03, respectively. Although there was no direct risk to human health from NOR and OFL in the Yitong River in Changchun based on this assessment, the physical health risk to children was slightly higher than that to adults. In a study conducted in the eastern part of China on 18 types of antibiotics, it was found that 58.3% of the urine samples from more than 1000 children had antibiotic residues^55^. Long-term exposure to antibiotics, especially in preschoolers, increases the risk for childhood obesity^56^.

**Figure 7:**
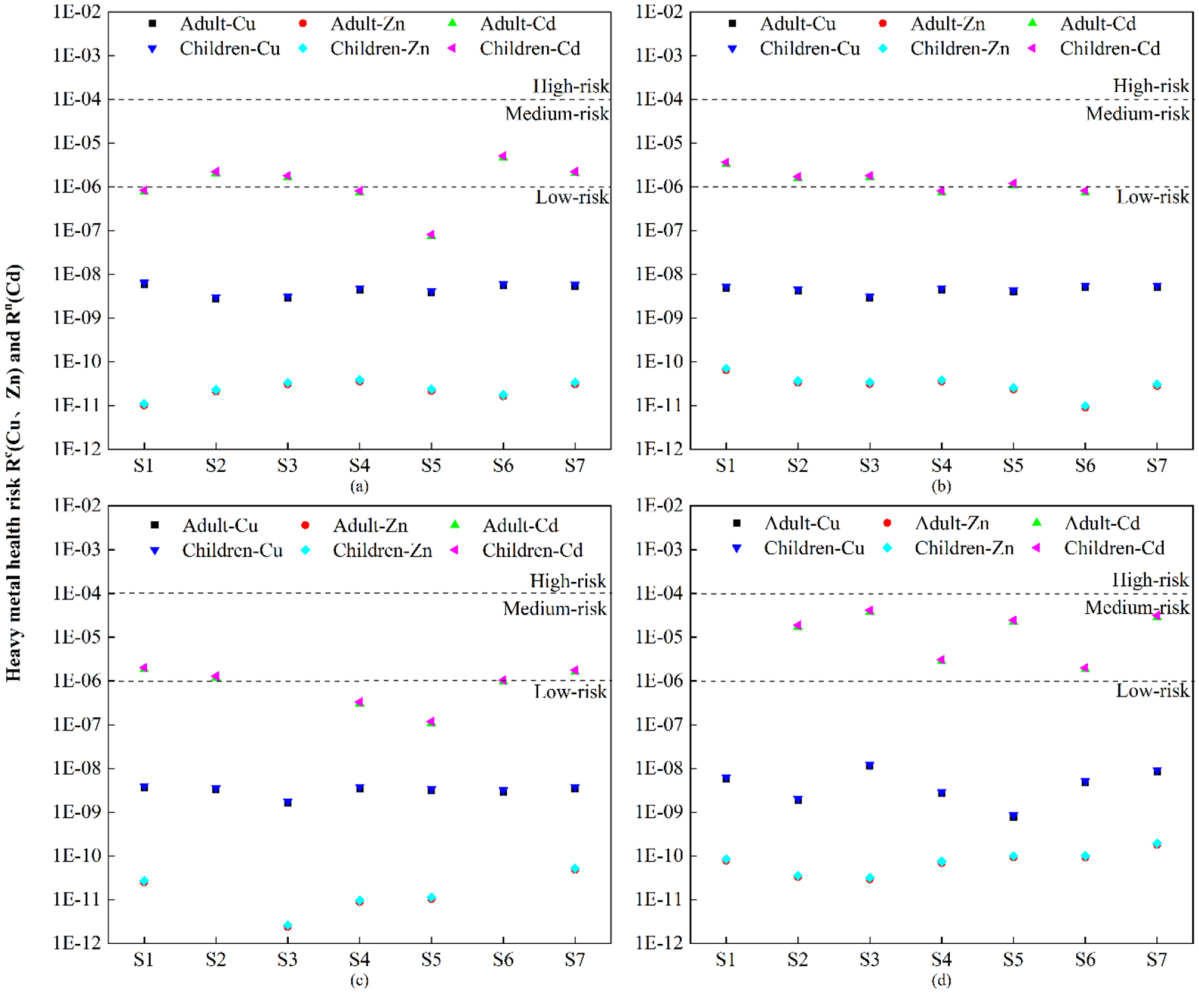
Risk assessment of heavy metal human health in Yitong River, Changchun. (**a**–**d**) are health risk assessments of heavy metals for adults and children in June, August, October and December, respectively.

Moreover, the R^c^ and R^n^ values for heavy metals in the Yitong River of Changchun for adult and child health risks were determined, and these are shown in Fig. 7. The Rc values of the noncarcinogenic heavy metals Cu and Zn were both at low risk levels, with no direct risk to adult health. The Rn value for the carcinogenic heavy metal Cd was at moderate and low risk levels, where the moderate risk rates by month were 57.14% in June, 71.43% in August, 57.14% in October, and 85.71% in December, with Cd being the most hazardous to adult health in winter. In terms of the spatial distribution characteristics, the order of Rn means at each point was in the following order from low to high: S4 (1.151E−06) < S1 (1.977E−06) < S6 (2.057E−06) < S5 (5.912E−06) < S7 (1.083E−05) < S3 (1.372E−05), which was at a medium risk level for adult health risk. The mean values for the entropy of noncarcinogenic health risk Rc for Cu for adults and children were 4.223E−09 and 4.610E−09, respectively, the mean values of the entropy of noncarcinogenic health risk Rc for Zn for adults and children were 4.057E−11 and 4.43E−11, respectively, and the mean values of the entropy of carcinogenic risk Rn for Cd for adults and children were 5.522E−06 and 6.02E−06. Heavy metals in the Yitong River in Changchun were at a low human health risk level, but the risk to children was higher than that to adults.

#### Mixed antibiotic-heavy metal toxicity assessment

Mixed antibiotic–heavy metal toxicity was evaluated according to the combined toxicity unit method and summation index method, and the results are shown in Supplemental Table 5. NOR-Cu, NOR-Zn, NOR-Cd, OFL-Cu, OFL-Zn and OFL-Cd were all antagonistic. This potentially occurred because the concentrations of the heavy metals Cu, Zn and Cd in the water column were much greater than the antibiotic NOR and OFL concentrations, resulting in an AI less than 0, which made them less toxic. The toxic effects of the combination of antibiotics and different heavy metals also differed, and the AI values of the combination of NOR with Cu, Zn and Cd were in the following descending order: NOR-Cu (− 1.866) < NOR-Zn (− 1.547) < NOR-Cd (− 1.028). The AI values for the combined toxicity of OFL with Cu, Zn and Cd were in the following order from lowest to highest: OFL-Cu (− 1.856) < OFL-Zn (− 1.554) < OFL-Cd (− 1.025). This phenomenon may have occurred because Cd is more toxic to Microcystis aeruginosa than Cu and Zn, and the more toxic substances make a greater contribution to combined toxicity.

In summary, antibiotics and heavy metals separately pose certain risks to the ecological environment. Individual toxicity poses low risks to human health, while mixed antibiotic-heavy metal toxicity is antagonistic, and the intensity of the toxic effects produced by the mixture is lower than that of any single substance. However, because long-term residues of antibiotics and heavy metals are present in the environment, antibiotic–heavy metal complexes can form. The fertile land in the northeastern plains of China has now become a well-known grain production area and livestock base in the north; thus, it is important to regulate antibiotics and heavy metals and continue research on these environmental pollutants to protect human health and the environment in China.

## Conclusion

In this study, we investigated the spatial and temporal distribution patterns of antibiotics and heavy metals in the Yitong River basin of Changchun, evaluated the effects of both types of pollutants on the ecological environment and human health and determined the toxicity of mixed antibiotic-heavy metal pollution. The concentrations of antibiotics detected showed a spatial and temporal distribution, with greater concentrations in summer than in winter, and greater concentrations in urban than in suburban areas, suggesting that anthropogenic pressures and different climatic conditions can contribute to antibiotic residues. Further research is needed to assess the impact of different environmental climates and human activities on antibiotic enrichment in urban environments. NOR and OFL showed medium and high ecological risk levels (RQ ≥ 0.1) in water bodies, no contamination levels (P_n_ ≤ 0.7) for the heavy metals Cu and Zn in the environment, and a low contamination level (0.7 ≤ P_n_ ≤ 1) for Cd. This may be due to the "detoxification" effect that occurs when high concentrations of heavy metals are present in a water column with low concentrations of antibiotics, which reduces the toxicity of the antibiotic-heavy metal mixture in the water column. Long-term residues of antibiotics and heavy metals and interactions between these two types of pollutants make pollution effects more complex and uncertain and increase the risks associated with pollution.

## Supplementary Information

Supplementary Information.

## Data Availability

The datasets used and/or analyzed during the current study are available from the corresponding author on reasonable request.
