# Supplementary material for: Spatial and temporal distribution characteristics of antibiotics and heavy metals in the Yitong River basin and ecological risk assessment
Source: Sci Rep. 2023 Mar 14;13:4202. doi: 10.1038/s41598-023-31471-5 (PMC10015007; doi:10.1038/s41598-023-31471-5)
Supplement: Supplementary file 1 — Supplementary Information. [file 41598_2023_31471_MOESM1_ESM.docx]

**Supplementary file**

**Supplemental Table 1 The parameters of Multiple-Reaction Monitorin**

**Supplemental Table 2. Antibiotics and heavy metals E (L) C_50_ parameters takeni ^[1]^ ^[2]^.**

**Supplemental Table 3. Quality elements and physico-chemical quality standards for assessment of ecological status of surface water in Romania, 2006 (GD 161).**

**Supplemental Table 4. Average weight of adults and children and daily water intake ^[3]^.**

**Supplemental Table 5. Joint Toxicity of Antibiotics and Heavy Metals to Microcystis aeruginosa.**

**Supplemental Figure 1. Water quality condition.** (a) (b) (c) and (d) are the water quality conditions in June, August, October and December, respectively.

**Supplemental Figure 2. Comparison of antibiotic and heavy metal levels in the environment Values with superscript letters a b are significantly different across columns (P＜0.05).**

**References**

**Supplemental Table 1. The parameters of Multiple-Reaction Monitorin**

| Antibiotics | CAS No. | MRM(DA) | DP (volts) | CE (volts) | RT (min) |
| --- | --- | --- | --- | --- | --- |
| Norfloxacin | 70458-96-7 | 320.3~302.1*320.3~276.0 | 37/36 | 30/27 | 5.06 |
| Ofloxacin | 82419-36-1 | 362.0~318.0*362.0~261.0 | 52/55 | 30/38 | 4.95 |

**Supplemental Table 2. Antibiotics and heavy metals E (L) C_50_ parameters takeni ^1^ ^2^**

| Metal | Organism | L(E)C_50_(mg/L) |
| --- | --- | --- |
| NOR | Microcystis aeruginosa | 0.062 |
| OFL | Microcystis aeruginosa | 0.021 |
| Zn | Microcystis aeruginosa | 0.08 |
| Cu | Microcystis aeruginosa | 0.09 |
| Cd | Microcystis aeruginosa | 0.10 |

**Supplemental Table 3. Quality elements and physico-chemical quality standards for assessment of ecological status of surface water in Romania, 2006 (GD 161)**

|  | Class I | Class II | Class III | Class IV | Class V |
| --- | --- | --- | --- | --- | --- |
| DO(mg/l) | 9 | 7 | 5 | 4 | >4 |
| Total P(mg/l) | 0.15 | 0.4 | 0.75 | 1.2 | >1.2 |
| Total N (mg/l) | 1.5 | 7 | 12 | 16 | >16 |
| CODcr(mg/l) | 10 | 25 | 50 | 125 | >125 |
| Cadmium μg/l) | 0.5 | 1 | 2 | 5 | >5 |
| Copper(μg/1) | 20 | 30 | 50 | 100 | >100 |
| Zinc(μg/1) | 100 | 200 | 500 | 1000 | >1000 |

**Supplemental Table 4. Average weight of adults and children and daily water intake ^3^**

| Research Subjects | Gender | BW/kg | DWI/(L/d) |
| --- | --- | --- | --- |
| Children | Male | 24 | 0.81 |
|  | Female | 23 | 0.76 |
| Adults | Male | 66.1 | 2.48 |
|  | Female | 57.8 | 2.12 |

**Supplemental Table 5. Joint Toxicity of Antibiotics and Heavy Metals to Microcystis aeruginosa**

| Mixture | AI and 95% confidence interval | Mixture | AI and 95% confidence interval |
| --- | --- | --- | --- |
| NOR-Cu | -1.866（-2.162 ~ -1.571） | OFL-Cu | -1.856（-2.156 ~ -1.556） |
| NOR-Zn | -1.547（-1.847 ~ -1.248） | OFL-Zn | -1.554（-1.861 ~ -1.246） |
| NOR-Cd | -1.028（-1.042 ~ -1.014） | OFL-Cd | -1.025（-1.040 ~ -1.009） |

AI=0, the combined action of the mixture is additive; when AI<0, the combined action of the mixture is antagonistic; when AI>0, the combined action of the mixture is synergistic

**Supplemental Figures:**


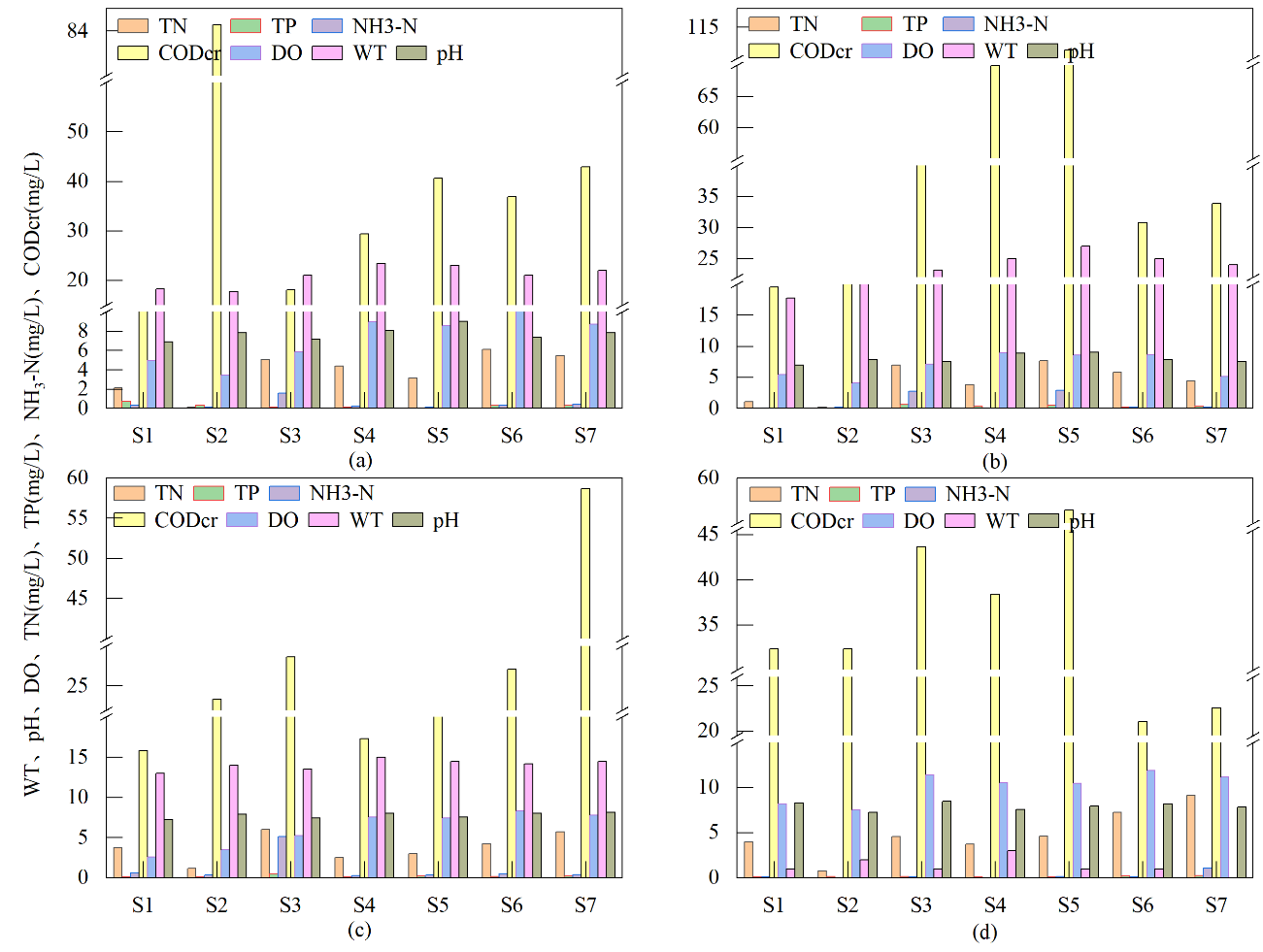


**Supplemental Figure1. Water quality condition**(a) (b) (c) and (d) are the water quality conditions in June, August, October and December, respectively


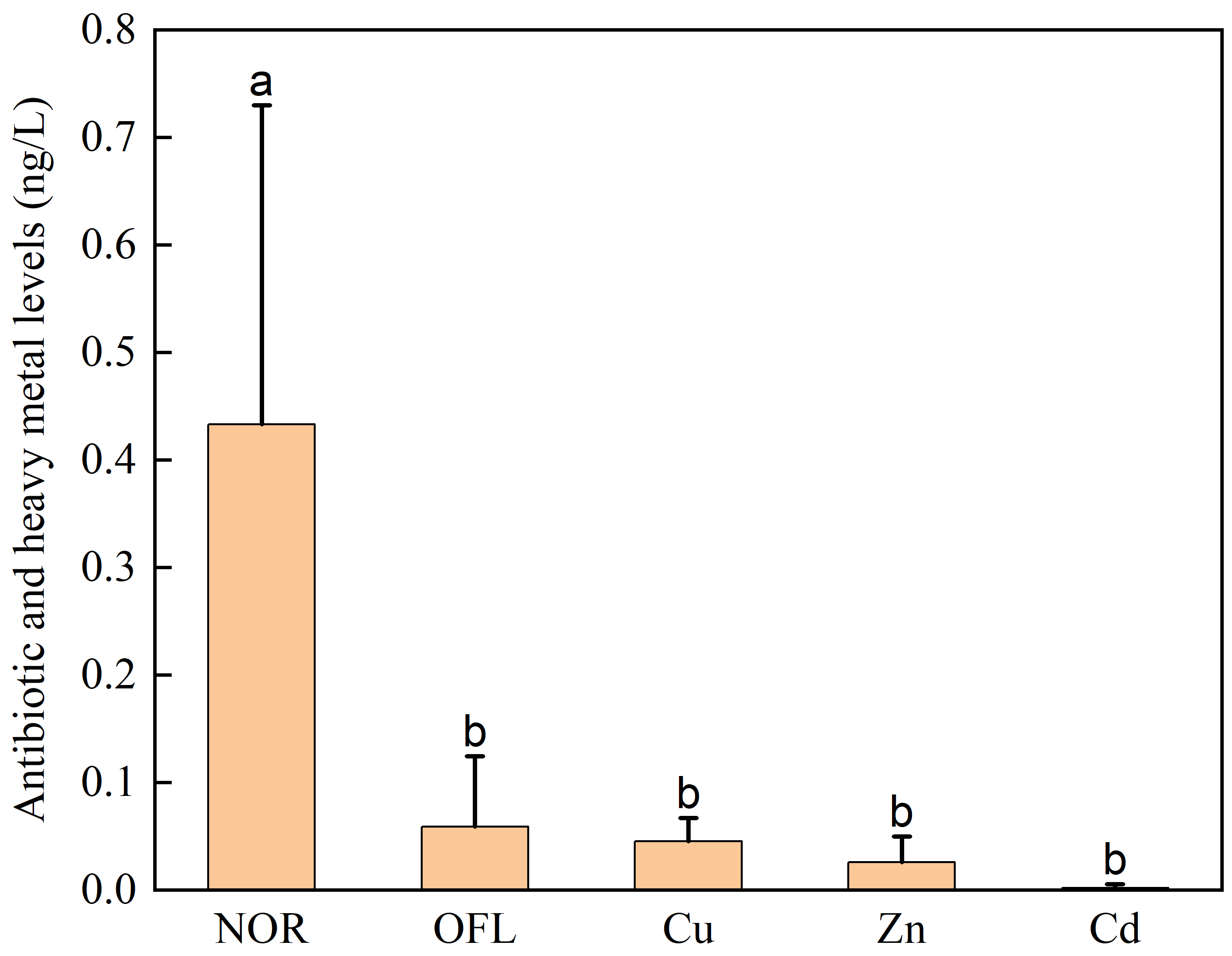


**Supplemental Figure2. Comparison of antibiotic and heavy metal levels in the environment**

**Values with superscript letters a b are significantly different across columns (P＜0.05)**

**References**

1 ZHAO Xinyu *et al.* Spatial and temporal distribution of quinolone antibiotics in soils of Shijiazhuang and its risk assessment *Environmental Science & Technology*, 1-17, doi:<https://doi.org/10.13227/j.hjkx.202204266> (2022).

2 Cheng, G. *Evaluation of the mixed toxicity of heavy metals and antibiotics to Microcystis aeruginosa*, Northwest Agriculture and Forestry University, (2020).

3 Shi, W. *et al.* Identification of trace organic pollutants in freshwater sources in Eastern China and estimation of their associated human health risks. *Ecotoxicology* **20**, 1099-1106, doi:<https://doi.org/10.1007/s10646-011-0671-8> (2011).
